# Dr. Koch

**Published:** 1891-03

**Authors:** 


### Dr. Koch.

Dr. Koch is 47 years old. After graduating at the Univeristy of Gottingen, he commenced practice in a little village near Hanover, but failed to make a living. He then tried Rackwitz, a small malarious town in Prussian Poland, with no better results. Finally he settled in Wollstein, and in 1880 he attracted much attention by his analyses and medical testimony in the famous Speichert poisoning case. In 1882 he discovered the bacillus of tuberculosis, and in 1883 the germ of cholera while acting as the head of the medical commission sent by the German Government to Egypt and India to study the causes and prevention of cholera. On his return to Germany he received an honorarium of 100,000 marks, the rank of Privy Councillor, and the Rectorship of the Imperial Institute of Hygiene.

